# The Expression of Hsa-Mir-1225-5p Limits the Aggressive Biological Behaviour of Luminal Breast Cancer Cell Lines

**DOI:** 10.2174/0122115366268128231201054005

**Published:** 2024-01-09

**Authors:** Y-Andrés Hernandez, Janeth Gonzalez, Reggie Garcia, Andrés Aristizabal-Pachón

**Affiliations:** 1 Departamento de Nutrición y Bioquímica, Facultad de Ciencias, Pontificia Universidad Javeriana, Bogotá D.C., Colombia;; 2 Departamento de Ciencias Fisiológicas, Facultad de Medicina, Pontificia Universidad Javeriana, Bogotá D.C., Colombia

**Keywords:** Breast cancer, microRNAs, tumor progression, invasion, migration, cancer proliferation

## Abstract

**Introduction:**

Numerous genetic and biological processes have been linked to the function of microRNAs (miRNAs), which regulate gene expression by targeting messenger RNA (mRNA). It is commonly acknowledged that miRNAs play a role in the development of disease and the embryology of mammals.

**Methods:**

To further understand its function in the oncogenic process, the expression of the miRNA profile in cancer has been investigated. Despite being referred to as a noteworthy miRNA in cancer, it is unknown whether hsa-miR-1225-5p plays a part in the *in vitro* progression of the luminal A and luminal B subtypes of breast cancer. We proposed that a synthetic hsa-miR-1225-5p molecule be expressed in breast cancer cell lines and its activity be evaluated with the aim of studying its function in the development of luminal breast cancer. In terms of the typical cancer progression stages, such as proliferation, survival, migration, and invasion, we investigated the role of hsa-miR-1225-5p in luminal A and B breast cancer cell lines.

**Results:**

Additionally, using bioinformatics databases, we thoroughly explored the target score-based prediction of miRNA-mRNA interaction. Our study showed that the expression of miR-1225-5p significantly inhibited the *in vitro* growth of luminal A and B breast cancer cell lines.

**Conclusion:**

The results were supported by a bioinformatic analysis and a detailed gene network that boosts the activation of signaling pathways required for cancer progression.

## 1 INTRODUCTION

Breast cancer is a relevant pathology that affects mostly women, with significant data worldwide related to incidence and prevalence. Several different types of breast cells are present in the core tumor mass and stromal components of breast cancer. Based on molecular and morphological characteristics, breast cancer has been categorized according to its aggressiveness due to tumor heterogeneity, mutational tumor burden, and response to various antitumoral drugs, which provides particular information about the likely tumor growth [1], [2]. Luminal breast cancer subtype is recognized for its better prognosis and better response to classical therapeutic strategies, specifically luminal A with a population incidence higher than 30% and based on St. Gallen expert consensus, its molecular classification panel is estrogen receptor (ER) positive, progesterone receptor (PR) ≥20%, HER2 negative and Ki67 < 14%. Luminal B, representing approximately 20% of breast female cancer [3], has been characterized by HER2+/-, ER+, PR negative or < 20% and Ki67 ≥20% [4]. Tumor behavior is composed of typical cell responses to stimuli and signals of the microenvironment that drives tumor cells to be able to increase, survive, migrate, and invade local and distant tissues. Each step of the tumor decision is a key phase of the tumor progression timeline that finally results in metastasis and poor prognosis. Cancer cell abilities are finely regulated by several molecules, all of which promote an equilibrium between antitumor or promoting tumor, that allows tumor cells to achieve their goal. At this point, microRNAs (miRNAs) described as short, non-coding RNA molecules have been remarkably associated with cancer development, which main role is to regulate the expression of several genes that have been reported as overexpressed or not expressed in cancer types, this phenomenon can be explained by the expression or not of miRNAs in specific tumor tissues [5]. In healthy conditions, miRNAs have specific activity that confers to cells capacities to differentiate, self-regulate, and intimately control cell proliferation with the purpose of avoiding pathological tissue conditions. Aligned with this theory, the trophoblastic model of cancer proposed by Beard *et al*., and revised by others [6] drives to review the placental development and its parallel with the tumor development, based on the theory that resumes the placental behavior as a pseudotumor. miRNAs knowledge has oriented and promoted the construction of tissue expression profiles that help to associate the role of these molecules in organ development. The miRNAs expression in the placenta has been reported, particularly the overexpression of some miRNAs that keep a significant expression level in different stages of healthy pregnancy. Into the group of placental miRNAs, hsa-miR-1225-5p was reported as significantly expressed, and its participation in placenta development was defined as crucial [7]. Due to the trophoblasts in the placenta's capacity for proliferation, survival, migration, and invasion, the placenta's behavior as a “pseudotumor” creates an intriguing opportunity to compare the expression of miRNAs in tumor cells and healthy placenta. [8]. The hsa-miR-1225-5p has been reported as a tumor inhibitor in other cancer models like pancreatic [9] and osteosarcoma [10], but information about the role of miR-1225-5p in breast cancer is unknown.

Considering the aforementioned, we suggested investigating the effect of hsa-miR-1225-5p on luminal A and B breast cancer cells' abilities to proliferate, survive, migrate, and invade. We also conducted a thorough bioinformatics study based on the mechanism utilized by miRNAs to identify the targets of hsa-miR-1225-5p and its potential routes to counteract the effects of the exogenous miRNA.

## 2 MATERIALS AND METHODS

### 2.1 Cell Culture

The American Type Culture Collection (ATCC^®^) released the human MCF-7 (luminal A) and BT-474 (luminal B) cell lines, which were then cultured in Dulbecco's modified Eagle's medium (DMEM; Lonza; Walkersville, USA) with 10% fetal bovine serum (FBS; Eurobio; Paris, France). Standard conditions for cell culture incubation were a humidified environment with 5% CO_2_ at 37°C.

### 2.2 Transient Transfection

MCF-7 and BT-474 cell lines were transfected in 6-well plates with the mix of hsa-miR-1225-5p mirVana mimic^®^ mature sequence AAACCGUUACCAUUACUGAGUU in 1500 μL of Opti-MEM^®^ (ThermoFisher Scientific, Waltham, USA), (ThermoFisher Scientific, Waltham, USA), after that, mix was incorporated with lipofectamine 2000^®^ (Invitrogen, Carlsbad, CA, USA). The transfection mixture was placed onto the cells. Cell plates were cultured for 24 hours under standard conditions. Twenty-four hours after transfection, the culture medium was discarded, and 1500 µL of DMEM-based cell culture medium was added. The following assays were conducted using transfected cells. All tests used cell lines that had been transfected with a scramble sequence as a control.

### 2.3 RNA Extraction and Gene Expression

After removing the media for the cell culture, the plates were cleaned with PBS 10X to confirm the transfection success. Following the manufacturer recommendations, TRIzol^®^ (ThermoFisher Scientific, Waltham, USA) was used for cell lysis and RNA extraction. In Nanodrop^®^ (ThermoFisher Scientific, Waltham, USA), the extracted RNA was quantified. Following that, RNA samples were processed using DNAse I (New England Biolabs, Massachusetts, USA) and TaqMan advanced miRNA cDNA synthesis kit^®^ (ThermoFisher Scientific, Waltham, USA) to generate cDNA. TaqMan miRNA advanced assays^®^ (ThermoFisher Scientific, Waltham, USA) were used to perform qPCR. The housekeeping gene used in the assay was GAPDH forward 5-3 GTCTCCTCTGACTTC AACAGCG, reverse 5-3 ACCACCCTGTTGCTGTAGCCA A. Finally, qPCR was used to amplify the targets from the RNA samples for the predicted miRNA-mRNA target score in genes: LASP1 forward 5-3 CTTCGCCTCAAGCAACAG AGTG, reverse 5-3 TGTCTGCCACTACGCTGAAACC, 3´ UTR sequence GUACCCAC; MYC forward 5-3 CCTGGTG CTCCATGAGGAGAC, reverse 5-3 CAGACTCTGACCTT TTGCCAGG, 3´UTR UUCCUUC and PEG10 forward 5-3 ACCACCAGGTAGATCCAACCGA, reverse 5-3 TGTCAG CGTAGTGACCTCCTGT, 3´UTR sequence GUACCCAA. RNA extracted from healthy placenta samples was used to evaluate the expression of selected genes as a control reference. Ethical and informed consent was obtained from previous research availed and approved by Instituto de Genética Humana of Pontificia Universidad Javeriana.

### 2.4 Colony Formation Assays

MCF-7 and BT-474 cells were seeded into 6-well plates at a density of 500 cells per well and grown for 10 days under standard conditions in accordance with the clonogenic *in vitro* assay technique [11]. The cells were then fixed and stained using Giemsa (Sigma Aldrich, United States). The colony formation rate was calculated using ImageJ software from the National Institutes of Health (Bethesda, MD, USA).

### 2.5 Proliferation Cell Assays

2.5x10^3^ cells of the MCF-7 and BT-474 cell lines were plated onto 96-well plates. Reading points were established using CytoPainter Cell Proliferation Staining Reagent-Green fluorescence (ABCAM, Cambridge, UK) at 4 hours, 24 hours, 48 hours, 72 hours, and 96 hours after transfection. Cell proliferation was measured using a FLUOstar Omega plate reader (BMG Labtech, Offenburg, Germany) and a fluorescence change at a wavelength of Ex/Em = 485/520.

### 2.6 Migration and Invasion Assays

Using 24-well plates with Thincert^®^ cell culture inserts (Greiner Bio-one, Monroe, USA), migration tests were carried out. Each culture well contained DMEM without serum and was at the top of the well. MCF-7 and BT-474, 5.0x10^4^ cells were placed in the upper side of the matrix well. The bottom of the well contained DMEM supplemented with 10% FBS to promote migration, which was divided by a migration membrane. Cells remaining in the top well were removed using a cotton swab after the membrane insert had been incubated for 12 hours, and the migratory cells that had stuck to it were fixed and colored with Giemsa. The migration rate was calculated using ImageJ software from the National Institutes of Health in Bethesda, MD, US. Based on the Boyden chamber modified approach [12], the cell invasion tests were carried out utilizing 96-well plates covered with basement membrane extract and 8 µm pore diameter. According to the manufacturer's instructions, experiments were carried out in the Cell Invasion Assay Basement Membrane (ABCAM, Cambridge, UK). In DMEM without serum, 5.0 x 10^4^ MCF-7 and BT-474 cells were plated in the upper well of the invasion chamber. 10% FBS was added to DMEM in the lower well of the chamber to promote cell invasion. After the invading cells in the bottom well were stained with Giemsa stain and the remaining MCF-7 and BT-474 cells in the top well were washed, each well was then evaluated for fluorescence intensity using a FLUOstar Omega plate reader (BMG Labtech, Offenburg, Germany) at Ex/Em = 485/570nm. RFU (Relative Fluorescence Units) was obtained.

### 2.7 hsa-miR-1225-5p Target Interaction Prediction

Potential gene targets were sought to explain the results based on the findings about the hsa-miR-1225-5p functional involvement. The search was carried out using the MirPath TarBase v7.0 modules of the bioinformatics applications DianaTools and mirTarBase. All platforms used “hsa-miR-1225” as the search keyword. A list of the genes that hsa-miR-1225-5p interacts with was compiled from each platform. From this first list, the bioinformatics platform's filters for “strong evidence” and “less strong evidence” levels of interaction evidence were applied, and targets with interaction values larger than 75% were taken into consideration to create a second list of target genes. The impact on cellular processes relevant to tumor progression, the amount of evidence, and the body of scientific literature were then considered when filtering the microRNA-mRNA interaction. On the KEGG (Kyoto Encyclopedia of Genes and Genomes) platform, the chosen genes were then assessed. The prefix “map” was used in the search process. The signaling pathways were subsequently acquired in respect to each search criterion.

### 2.8 Statistical Analysis

Each experiment was run in triplicate three times. Both parametric and non-parametric tests were used, depending on the normality of the data. The mean standard error is displayed for each result. *A p* value less than 0.05 was considered significant.

## 3 RESULTS

### 3.1 hsa-miR-1225-5p Expression in MCF7 and BT474 Cell Lines

The hsa-miR-1225-5p expression levels in the breast cancer cell lines MCF7 and BT474 were evaluated using qPCR. Results revealed that neither of the two cell lines had any hsa-miR-1225-5p expression (Ct > 38), in contrast to placenta tissue (Ct mean = 19). The effectiveness of the complex hsa-miR-1225-5p and lipofectamine in transfected breast cancer cells was then verified using qPCR. The transfected group displayed an increased expression, with media values in fold change obtained: MCF7 transfected (Ct mean = 18) and BT474 transfected (Ct mean = 17), according to the results of contrasting exploration between control and hsa-miR-1225-5p transfected cells.

### 3.2 hsa-miR-1225-5p Reduces Proliferation and Survival Capacities in Luminal Breast Cancer Cells

**Fig. (1):**
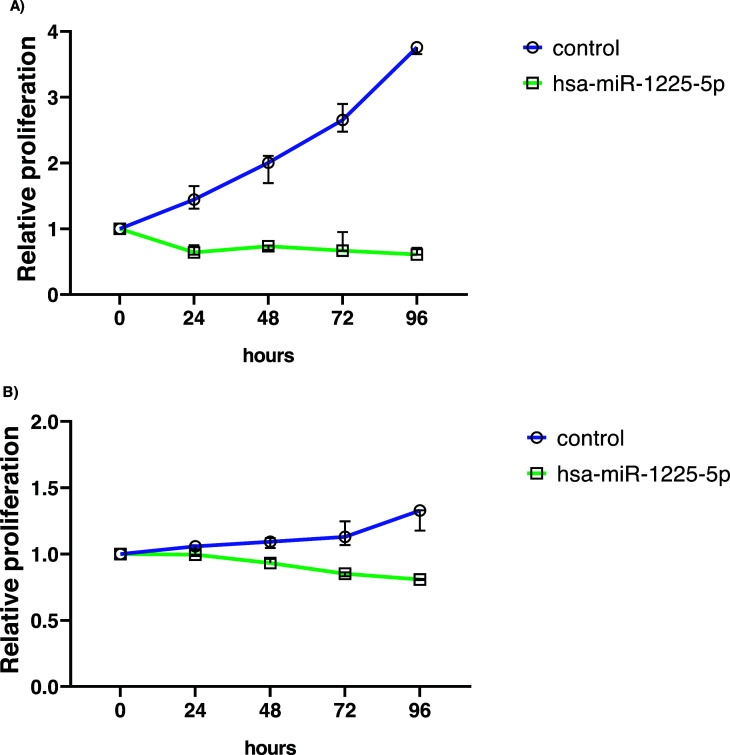
hsa-miR-1225-5p effects on proliferative abilities. (**A**). Proliferation assay for MCF7 cell line. (**B**). BT474 in proliferation assay. Control cells compared to cells transfected with hsa-miR-1225-5p. Statistical test two-way ANOVA.

On a timeline with 5 reading points, hsa-miR-1225-5p transfected in MCF7 cancer cells, showed proliferative abilities significantly inhibited (Fig. **1A**). Data analysis revealed statistically significant differences between transfected cells and control cells (*p* < 0,0001). The proliferative capacities of BT474 cells transfected with hsa-miR-1225-5p, which were maintained under the same growth conditions, were mostly diminished in comparison to control (*p* < 0,0001). In the BT474 cell line, the findings revealed consistent results about population doubling time (Fig. **1B**).

**Fig. (2):**
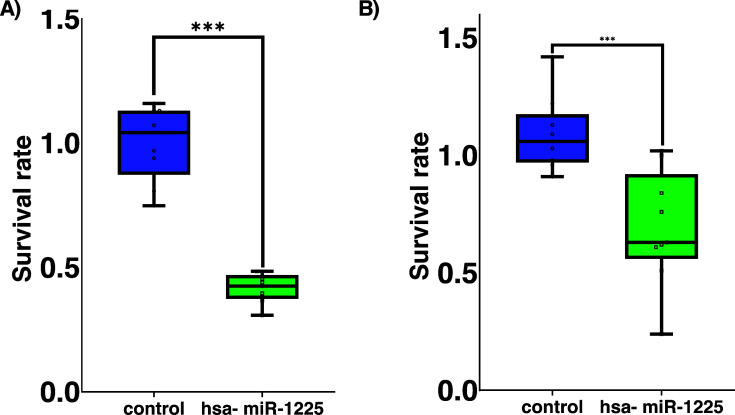
Breast cancer survival rate with expression of hsa-miR-1225-5p. (**A**). MCF7 cells showed reduced colony formation capacities related to control cells. (**B**). BT474 cancer cells revealed diminished colony forming abilities. Data evaluation of the BT474 behavior is consistent with proliferative features. Results of colony formation showed a substantially different value (*p* <0,0001). Statistical test t-student.

With regard to the survival assay, our results revealed a significant difference between the control and transfected cells (*p* < 0,0001), indicating that overexpression of hsa-miR-1225-5p affects cell survival and weakens the potential to form colonies *in vitro*, demonstrated as survival rate (SR). *In vitro* survival capacity of MCF7 (Fig. **2A**) and BT474 (Fig. **2B**) cancer cells were decreased significantly in comparison to the control group.

### 3.3 Migratory Capacities of Breast Cancer are Impaired by hsa-miR-1225-5p Expression

**Fig. (3):**
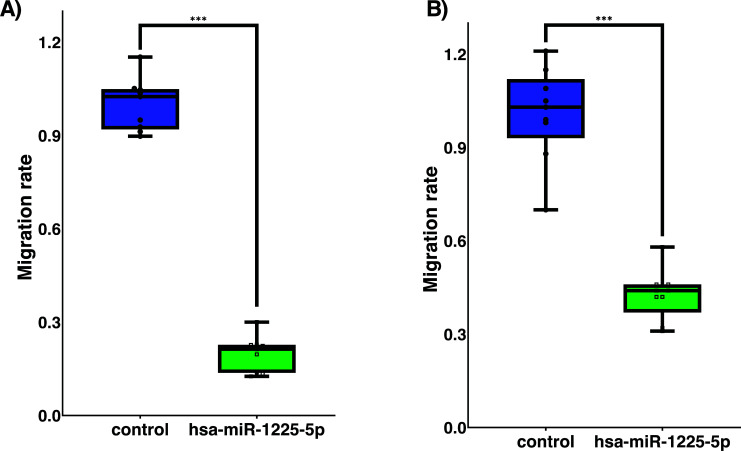
Migration capacities of luminal breast cancer cells. (**A**). MCF7 cells in migration assay; (**B**). BT474 cells in migration assay. Statistical test t-student.

A Boyden chamber method that had been adapted to test and evaluate MCF7 cells for controlled migration was used. The migratory abilities of MCF7 cells transfected with hsa-miR-1225-5p were substantially decreased (Fig. **3A**) compared to control group and showed that it was significantly reduced (*p* = 0.0001). Due to the expression of hsa-miR-1225-5p, BT474 cells showed a significantly decreased migratory ability (Fig. **3B**) as compared to control group. Both cancer cells behaved in a manner consistent with results of the migration assay (*p* = 0.0001).

### 3.4 The Invasion Potential of Breast Cancer Cells is Negatively Affected by hsa-miR-1225-5p Expression

**Fig. (4):**
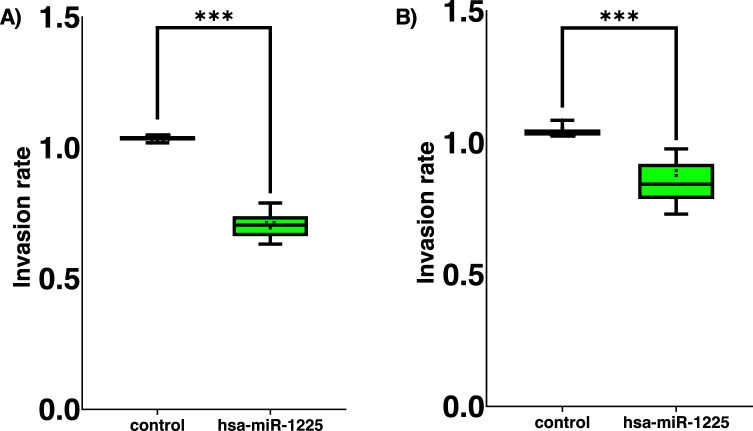
Cell invasion assay with or without expression of hsa-miR-1225-5p. (**A**) MCF7 cells transfected group compared to control cells; (**B**) BT474 cells transfected group compared to control cells. Statistical test t-student.

*In vitro* invasion capacities of MCF7 cells transfected with hsa-miR-1225-5p and control cells were assessed. We found that hsa-miR-1225-5p in MCF7 transfected cells showed significant reduced invasion potential (*p* = 0.001; Fig. **4A**). The behavior of BT474 cells was impressively consistent, with lower invasion properties in the transfected group than in the control cells, with a value of (*p* = 0.08152; Fig. **4B**).

### 3.5 Analysis of Targets for hsa-miR-1225-5p *In silico*

**Fig. (5):**
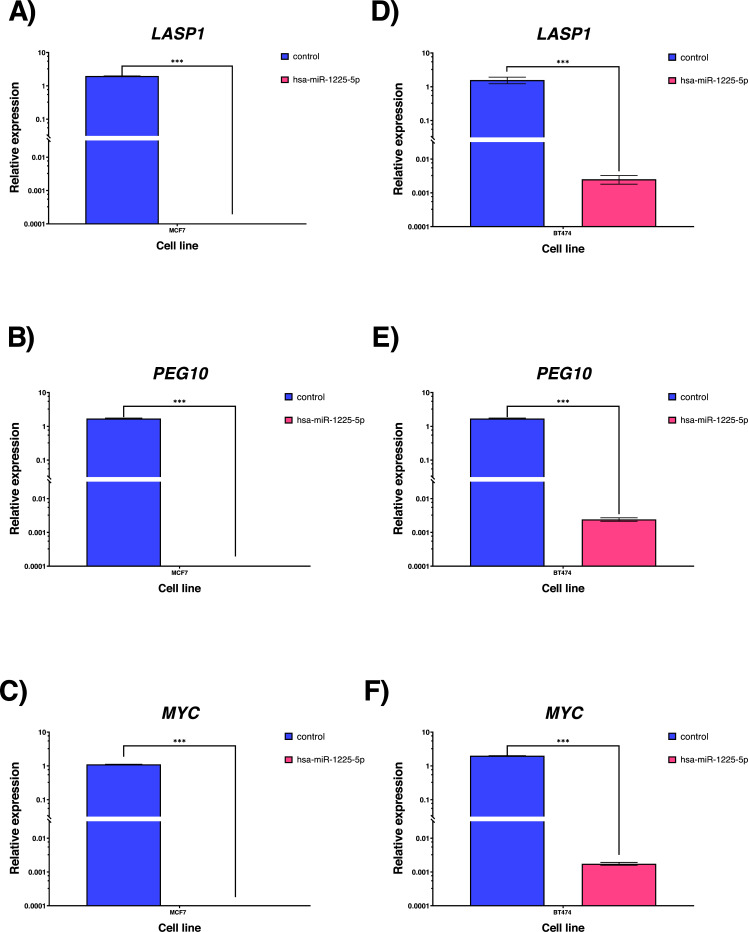
Relative expression of mRNA for hsa-miR-1225-5p targets. (**A**, **B**, **C**) Gene expression in MCF7 cell line. (**D**, **E**, **F**) Gene expression in BT474 cell line. Statistical test t-student.

The target genes for hsa-miR-1225-5p were found and evaluated using TarBase V.8 [13]. 213 target gene interactions with supporting evidence were found. The targets with the highest interaction scores and connections to pathways for cell viability or cancer progression were then chosen. We chose the gene targets based on these standards: *LASP1, PEG10* and *MYC*, all of them underwent *in vitro* qPCR analysis to measure mRNA expression in transfected and control cell lines. We found a significant decrease in the expression of the target genes when the hsa-miR-1225-5p was expressed Fig. **5**.

## 4 DISCUSSION

Because of the high incidence and fatality rates in the female world population, breast cancer is a public health concern. The metastatic possibility of cancer cells is linked to their capacities to proliferate, survive, migrate and invade tissues that finally drives to systemic organ failure [14]. Understanding the cancer biology allows us to comprehend the mechanisms by which different regulatory molecules, such as microRNAs, play a crucial part in the success of tumor progression, which can happen when these microRNAs are absent or overexpressed in tumors. The microRNAs have a fundamental role in the regulation of gene expression through the interaction with mRNA, which can subsequently be processed by the mRNA destabilization pathway or lead to degradation due to this microRNA-mRNA interaction [15]. In our study, we have found that microRNA-1225-5p was overexpressed in the placental tissue that we used as our reference tissue according to the report of Mouillet, *et al.* [16], but we subsequently found that it was not expressed at all in the luminal A and B cell lines. Based on our findings, we revealed that hsa-miR-1225-5p predominantly functions as an antitumor since it significantly reduces the abilities to proliferative, survive, migrate, and invade once expressed in mammary tumor cells evaluated. In accordance with the *in vitro* outcomes, we carried out a thorough bioinformatics analysis of the targets that the hsa-miR-1225-5p interacts with. Additional filters were then applied to increase the strength of the evidence that was available from public sources regard microRNAs. With the support of the bioinformatic analysis, we were able to determine 3 genes of interest based on the target score and their impact on cell signaling pathways involved in the development of tumors.

*LASP1* gene has relevant participation in signaling pathways that supports cell proliferation and cytoskeleton organization through PI3K/AKT/mTOR [17-19]. The participation of LASP1 in tumor development has been investigated in several biological models where its useful function as a mediator in pathways leads to tumor growth. Ruggieri, *et al*. strengthened *LASP1* activity and emphasized how this gene can be used to support a variety of tumor processes [20]. *LASP1* has been linked to bladder cancer tumor proliferation *via* interacting with the PI3K signaling pathway, according to research by Zhen *et al*, [21]. Also, the pro-tumoral activity of *LASP1* was studied by Wang *et al*. in a model of triple negative breast tumor cells and they reported that inhibiting *LASP1* with another microRNA (hsa-miR-203) dramatically reduced tumor migration [22]. Zheng *et al*. revealed *LASP1* pro-tumoral activity in a gastric cancer model, in which this gene suppression successfully decreased the tumor cells capacity for proliferation, invasion and migration [21]. Previous research has demonstrated that TGF-1 acts as an activator of *LASP1*, which favors the activation of AKT and, later, by phosphorylation, promotes the activity of the TSC1/TSC2 complex and stimulates mTORC1. This activation enables P70S6K, a powerful tumor promoter that has been specifically linked to breast cancers [23]. *LASP1* plays an essential role for the activation of the PI3K/AKT signaling pathway, which leads to in the overexpression of nuclear factors like E2F and induces proliferation and survival. The relationship between hsa-miR-1225-5p and *LASP1* is supported as a signaling axis that, once *LASP1* is blocked, the tumor-promoting activity described in multiple tumor models decreases, as previously discussed, and based on our results, silencing LASP1 gene by hsa-miR-1225-5p luminal A and B breast cancer cells significantly decreased proliferative potential. We found that migration abilities in luminal breast cancer cells were diminished in comparation with control cells. With the aim of assessing our results, the previously described search for the enrichment of cell signaling pathways and the prediction of targets was conducted. The interaction between hsa-miR-1225-5p and the *PEG10* gene (Paternally Expressed Imprinted Gene 10) was identified with a significant target predictive value. *PEG10* has been mainly associated with embryonic development and supports bone and cartilage-forming niche [24]. *PEG10* has been referred to in previous publications as a key regulator of cell migration and aggressive phenotype maintenance in tumor models, involving the participation of cell signaling intermediaries like the E2F family of transcriptional factors [25], which have been extensively characterized as significant players in cell cycle progression, cell migration and invasion processes [26]. Li, Xinran *et al*, reported the protumoral activity of *PEG10* gene in breast cancer cells and validated the relationship between aggressive breast cancer cells and overexpression of *PEG10* favoring the reorganization of cytoskeleton and enhanced migration capacities [27, 28]. Remarkably, PEG10 expression was evaluated in hepatocarcinoma tissues and was reported as a poor prognosis biomarker due to its properties to enhance the proliferation and migration of liver cancer cells and its role in liver tumor recurrence [26]. Overexpression of PEG10 in breast cancer cells is mediated by hyperactive status of receptor tyrosine kinases (RTKs), which are driven by external stimuli found within the tumor microenvironment, particularly transforming growth factor, activates RTKs and promotes the signaling cascade [29]. Due to interaction of hsa-miR-1225-5p and *PEG10* could be a plausible explanation for diminished migration properties of luminal breast cancer cells, highlighting that activation of *MYC*, which is coexpressed with *PEG10* in a wide range of cancers, is caused by the phosphorylation of signaling intermediaries like Smad2/3 and is simultaneously promoted by MYC activation. The coworking network between *PEG10* and *MYC* promotes that Smad2/3 and Snail are translocated into the nucleus [30], which is known to be a hallmark for the beginning and maintenance of tumor growth, survival and migration [31]. By interacting with hsa-miR-1225-5p, which is not present in breast tumor tissue, the negative regulation of *PEG10* is able to limit *PEG10* activity, which in turn results in negative regulation of the pathway activation. The *MYC* gene, which has been reported as the target of hsa-miR-1225-5p, was included to perform a comprehensive review of the results and the integrated interaction between cell signaling pathways based on bioinformatics analyses. *MYC* belongs to a group of genes that are activated by several mitogenic stimuli and promote a wide range of cellular processes from cell proliferation to oncogenic potential, being recognized as protooncogene [32, 33]. *MYC* activity is mainly described as an enhancer of tumor development [34]. In breast cancer, the expression and significative amplification of *MYC* are finely correlated with maintained tumor progression, and its transcriptional machinery is linked to aggressive cancer phenotypes with powerful abilities to invade local and distant tissues [35, 36]. In prostate cancer, Rebello *et al*, remarked the role of *MYC* as an oncogenic driver in advanced stages and the link between *MYC* expression and enhanced invasive abilities of cancer cells [37]. According to our findings in the MCF7 and BT474 cell lines, *MYC* activation is responsive to a variety of external stimuli, such as growth factors and hormones [38], for which, it integrates signaling pathways previously discussed in this work. Tyrosine kinase receptors are available, and once activated, they phosphorylate Ras [39] and can then activate one of two intracellular signaling pathways, PI3K/AKT [40] or MEK/MAPK [41]. These signaling pathways can then directly induce *MYC* activity and subsequently stimulate the activity of critical nuclear factors that govern tumor migration and invasion, of which the activity of E2F1, CDK4, p27, and p15 stands out [42-46]. Our results on the biological role of *MYC* in the development of breast luminal tumors and the many mechanisms of *MYC* activation enable us to consider the possibility of this gene as a potential target for effective anticancer treatments [47-49]. The invasive and migratory abilities of MCF7 and BT474, breast tumor cells, were reduced because of the negative regulation mediated by hsa-miR-1225-5p. Additional investigations have also supported the inhibition of *MYC* activity.

## CONCLUSION

Given their hyperactivation-promoting tumor abilities, the hsa-miR-1225-5p interaction targets *LASP1*, *PEG10*, and *MYC* that were chosen from curated open data have been thoroughly examined in tumor models with meaningful participation. We were able to demonstrate the antitumor function of hsa-miR-1225-5p in mammary tumor lines of the luminal subtype by the results of our experimental work and *in silico* research. The expression of hsa-miR-1225-5p negatively regulated the cell signaling pathways involved in the traditional processes of tumor progression, inhibiting the capacity of the tumor cells and significantly reducing tumor ability.

## Data Availability

All data obtained from curated public repositories in bioinformatics are available according to the references included in this work. Additional data can be obtained by express request to the main author.

## LIST OF ABBREVIATIONS

ER: Estrogen Receptor
miRNAs: microRNAs
PR: Progesterone Receptor
RTKs: Receptor Tyrosine Kinases
